# Extracellular vesicles as potential biomarkers and treatment options for liver failure: A systematic review up to March 2022

**DOI:** 10.3389/fimmu.2023.1116518

**Published:** 2023-02-22

**Authors:** Wang Lu, Huixin Tang, Shanshan Li, Li Bai, Yu Chen

**Affiliations:** ^1^ Fourth Department of Liver Disease, Beijing Youan Hospital, Capital Medical University, Beijing, China; ^2^ Beijing Municipal Key Laboratory of Liver Failure and Artificial Liver Treatment Research, Beijing, China

**Keywords:** extracellular vesicles, liver failure, diagnosis, prognosis assessment, treatment, systematic review

## Abstract

**Introduction:**

Extracellular vesicles (EVs) carrying functional cargoes are emerging as biomarkers and treatment strategies in multiple liver diseases. Nevertheless, the potential of EVs in liver failure remains indistinct. In this systematic review, we comprehensively analyzed the potential of EVs as biomarkers of liver failure and the therapeutic effects and possible mechanisms of EVs for liver failure.

**Methods:**

We conducted a systematic review by comprehensively searching the following electronic databases: PubMed, Web of Science, Embase and Cochrane Central Register of Controlled Trials from inception to March 2022. The used text words (synonyms and word variations) and database-specific subject headings included “Extracellular Vesicles”, “Exosomes”, “Liver Failure”, “Liver Injury”, etc.

**Results:**

A total of 1479 studies were identified. After removing 680 duplicate studies and 742 irrelevant studies, 57 studies were finally retained and analyzed. Fourteen studies revealed EVs with functional cargoes could be used to make the diagnosis of liver failure and provide clues for early warning and prognostic assessment of patients with liver failure. Forty-three studies confirmed the administration of EVs from different sources alleviated hepatic damage and improved survival through inhibiting inflammatory response, oxidative stress as well as apoptosis or promoting hepatocyte regeneration and autophagy.

**Conclusions:**

EVs and their cargoes can be used not only as superior biomarkers of early warning, early diagnosis and prognostic assessments for liver failure, but also as potentially effective treatment options for liver failure. In the future, large-scale studies are urgently needed to verify the diagnostic, predictive and therapeutic value of EVs for liver failure.

## 1 Introduction

Liver failure is a severe liver disease syndrome caused by multiple precipitating factors, which accompanies by grievous liver dysfunction or decompensation. The typical clinical manifestations of this syndrome include jaundice, coagulation dysfunction, ascites, hepatorenal syndrome, and hepatic encephalopathy (1, 2). At present, effective treatment for liver failure is lacking except for liver transplantation, although it still faces challenges of graft rejection, high cost and donor shortage. The transplant-free survival in patients with liver failure is rather low, usually less than 50% (3–6). Especially, diverse etiologies, complex clinical manifestations, undefined pathogenesis, high mortality and lack of effective treatments make it more difficult to accurately diagnose and properly treat patients with liver failure. In this context, it is extremely important to seek candidate biomarkers for early warning, early diagnosis and prognostic assessment of liver failure (7, 8). On the other hand, exploring potentially effective therapies for liver failure is also essential for improving survival rate, which is the scientists have been working on all the time (9–11).

Extracellular vesicles (EVs) are nanoscale vesicles with proteolipid bilayers. They can be secreted by almost all cells into the extracellular milieu, which makes them widely distributed in various biological fluids, such as blood, urine, milk, sputum, ascites, and cerebrospinal fluid (12). EVs are broadly divided into two main subgroups depending on their biogenesis: exosomes (Exos, 50-150 nm in diameter) and microvesicles or microparticles (MVs or MPs, 50-500 nm in diameter, up to 1000 nm) (13, 14). EVs have been considered metabolic waste. Over the past decade, scientists have demonstrated that EVs contain various bioactive substances or signal transduction molecules that exert crucial roles in homeostasis maintenance, antigen presentation, gene regulation, and so on (15, 16). Considering that EVs can reflect the pathophysiological state of the cells from which they are derived, EVs have the great potential to emerge as non-invasive biomarkers for early diagnosis and prognosis assessment of liver diseases (17–21). In addition, EVs possess the following advantages: stable membrane structure, low immunogenicity, good histocompatibility, easy chemical and genetic programming. Therefore, it is feasible to utilize EVs as drugs or drug carriers to treat refractory diseases such as cancer, neurological and cardiovascular disorders (22–24).

So far, there isn’t a universal ideal animal model for liver failure in view of multiple etiologies and complex mechanisms related to this syndrome (25, 26). Currently available animal models of liver failure have their strengths and weaknesses. Animal models of acute liver failure (ALF) enrolled in the present systematic review mainly involve hepatotoxic drug-induced, surgically induced, and mixed models. Virus-induced ALF models are rare and unsatisfactory (27). The commonly used drugs and chemical reagents for ALF induction include D-galactosamine (D-GalN) with or without lipopolysaccharide (LPS) or tumor necrosis factor-α (TNF-α), acetaminophen (APAP), carbon tetrachloride (CCl_4_), concanavalin A (Con A) and thioacetamide (TAA) (28, 29). The surgical ALF models can be divided into hepatectomy (total or partial), devascularization (total or partial) and a combination of both (30, 31). Hepatic ischemia-reperfusion injury (IRI), as an inevitable local sterile inflammatory response following surgery, is one of the main causes of early organ dysfunction and failure after liver transplantation (32). And hepatic IRI model accounts for a large proportion of surgically induced ALF models. Animal models of acute-on-chronic liver failure (ACLF) are usually induced by acute insult [such as LPS and/or D-GaIN, or ethyl alcohol (EtOH)] in the setting of chronic liver injury (for example, CCl_4_ or bile duct ligation) (33).

Till now, studies on EVs as biomarkers or therapeutic options for liver failure are still in the infancy. This systematic review summarized the current available studies including preclinical animal studies and clinical studies in this field, and hopes to provide novel ideas and directions for the early warning, diagnosis, treatment and prognostic assessment of liver failure.

## 2 Methods

Our systematic review was prepared according to the Cochrane recommendations for study methodology and the Preferred Reporting Items for Systematic Reviews and Meta-Analyses (PRISMA) guidelines (34).

### 2.1 Literature search strategy

The search strategy was developed by two informatics specialists. We comprehensively and systematically searched the following electronic databases: PubMed, Web of Science, Embase, and Cochrane Central Register of Controlled Trials from inception to March 2022. The used text words (synonyms and word variations) and database-specific subject headings included the followings: “Extracellular Vesicles”, “Exosome”, “Liver Failure”, “Liver Injury”, and so on. The search strategy for PubMed was summarized in **Table S1**, and it was also applicable to all other databases.

### 2.2 Eligibility criteria

We enrolled the studies according to the following criteria: (1) studies on EVs or modified EVs. (2) studies on preclinical or clinical studies of liver failure. (3) studies on EVs as potential biomarkers and treatment options for liver failure.

We excluded the studies according to the following criteria: (1) studies unrelated to EVs. (2) studies on the application of EVs in diseases other than liver failure and liver injury we described in the Introduction. (3) conference abstracts, reviews, mechanism studies, non-English articles and *in vitro* studies.

### 2.3 Data extraction

The following data were extracted from each study: experimental model, EV source, EV separation techniques, EV administration including dosage and route, EV cargoes, EV functions and others (See **Tables 1**–**6** for details). To select articles potentially eligible for inclusion, two authors independently screened the title and abstract of each article, then reviewed the full texts of all retaining studies and analyzed the related information. The divergence between these two authors was judged by the corresponding author. We did not conduct a meta-analysis due to the limited number of available randomized controlled trials. All included studies recruited patients that provided informed consent before enrollment.

**Table 1:** The potential biomarkers for liver failure/injury in animal studies.

Biomarker	Type of EVs	Source	Expression	Animal model	Description	Year	Ref.
Cd26, Cd81, Slc3A1	Exos-like urinary vesicles	Urine	Down	Wistar rats, D-GalN 1000 mg/kg/5mL i.p.	Decrease in urinary vesicles in D-GalN-induced ALI.	2010	(35)
Surface CD133^+^ and CD39^+^	MPs	Plasma	Up	C57BL/6, APAP 300 mg/kg i.p.	HSC and CD133^+^ MPs levels increase in a CD39-dependent manner during APAP-induced ALI.	2013	(36)
CD41^+^, Ly-6G^+^, CD62E^+^	MPs	Plasma	Up	C57BL/6, IRI model	MPs derived from platelets and neutrophils may be markers of inflammatory injury, and MPs derived from endothelial cells may be important in angiogenesis during the reparative phase.	2014	(37)
CES3, SLC27A2, HSP90, HSP70, FRIL1, CPS1, MAT, COMT;	EVs	Serum	Up	SD rats, D-GalN 1000 mg/kg/5mL i.p.	These protein expression levels of EVs increase in D-GalN-induced ALI through *in vivo* and *in vitro* experiments, which coincide with the proteomic data. Clusterin is drastically reduced by the treatment with D-GalN.	2014	(38)
clusterin			Down				
ALB, HP, FGB	EVs	Plasma	Up	Balb/C, APAP 300 mg/kg i.p. D-GalN 1000 mg/kg i.p. TAA 200 mg/kg i.p.	Upregulated in liver injury induced by APAP and reversed to basal levels by NAC or GSH. Similar results are observed in TAA or D-GalN-induced liver injury.	2017	(39)
miR-122, miR-192, miR-155	Exos	Plasma	Up	Balb/C, APAP 300 mg/kg i.p.	Upregulated in APAP-induced liver injury and reversed to basal levels by NAC.	2017	(40)
CYP2d1	EVs	Serum	Up	Wistar rats, GalN 1000 mg/kg/5mL i.p.	Show an increased activity in serum EVs after GalN-induced injury.	2018	(41)
miR-122a-5p, miR192-5p, miR193a-3p	Exos	Serum	Up	Wistar rats, APAP 1000 mg/kg i.p. TAA 1000 mg/kg i.p.	Exosomal miR-122a-5p shows higher diagnostic power and a wider diagnostic window in ALI. Exosomal miR-122a-5p, 192-5p and 193a-3p exhibit an injury-specific signature in ALI.	2018	(42)
miR-122-5p, miR-192-5p, miR-22-3p	EVs	Serum	Up	C57BL/6, (1) ALF: 10% CCl_4_ 0.5 mL/kg i.p.; (2) CLF: 10% CCl_4_ 0.5 mL/kg, twice a week for 8 wk i.p.	The differential serum EVs miRNAs in ALI are associated with liver steatosis and inflammation, while those in CLI are associated with HCC and hyperplasia. ALI serum EVs also change the phenotype of liver macrophages.	2021	(43)

ALB, albumin; ALF, acute liver failure; ALI, acute liver injury; APAP, acetaminophen; CCl_4_, carbon tetrachloride; CLF, chronic liver failure; CYP2d1, cytochrome P450 cytochrome 2d1; D-GalN, D-galactosamine; EVs, extracellular vesicles; Exos, exosomes; FGB, fibrinogen; GSH, glutathione; HCC, hepatocellular carcinoma; HP, haptoglobin; i.p., intraperitoneal injection; IRI, ischemia-reperfusion injury; MPs, Microparticles; NAC, N-acetylcysteine; SD, Sprague Dawley; TAA, thioacetamide.

### 2.4 Quality assessment

We used the Systematic Review Centre for Laboratory Animal Experimentation (SYRCLE) tool to evaluate the risk of bias for preclinical studies (94). And the risk of bias graph was drawn using the RevMan 5.3.2 software provided by the Cochrane Collaboration Network. The risk of bias for clinical studies was assessed using the Newcastle-Ottawa scale (NOS). The Confidence in the Evidence from Reviews of Qualitative research (CERQual) tool was used to assess the evidence quality of outcomes in this systematic review (95). The quality assessment was done independently by two authors and the divergence between these two authors was judged by the corresponding author. A PRISMA figure was created.

## 3 Results

### 3.1 Literature selection

**Figure 1:**
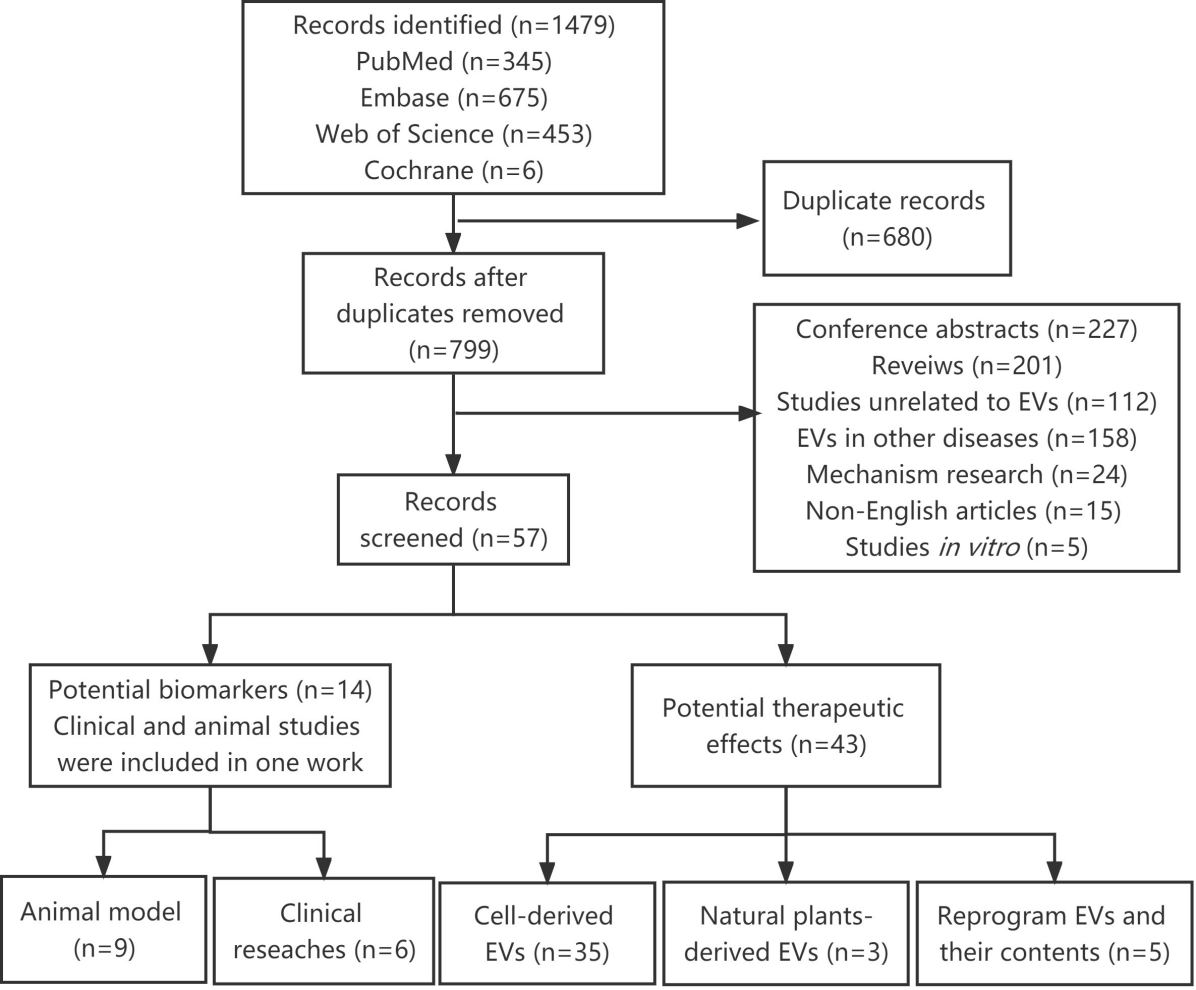
Flow diagram for the selection of studies.

A total of 1479 articles were identified using our search strategy. After removing 680 duplicates, 799 articles were submitted to the title, abstract or full-text assessment by two independent authors. Among these, 742 irrelevant articles were further excluded, including conference abstracts, reviews, studies unrelated to EVs, EVs in other diseases, mechanism research, non-English articles and *in vitro* studies. Finally, 57 articles were retained and analyzed in this systematic review (**Figure 1**).

### 3.2 EVs as potential biomarkers for early warning, diagnosis and prognostic assessment of liver failure

We summarized the studies on EVs as potential biomarkers of liver failure. **Tables 1**, **2** listed the relevant preclinical and clinical studies, respectively. This review revealed that EVs not only could be utilized as the early warning or diagnosis markers of liver failure, but also as biomarkers for prognostic assessment of this syndrome.

**Table 2:** The potential biomarkers for liver failure/injury in clinical studies.

Biomarker	Source	EVs separation	Patients (Country)	Se/Spe (%)	AUROC	Outcome	Year	Ref.
Surface CD133^+^ and CD39^+^	Plasma	Ultracentrifugation FACS	ALI: 5 Acute on chronic liver injury: 5 Controls: 7 (America)	n.a.	n.a.	Plasma CD39^+^ and CD133^+^ MPs levels increase in patients with acute or acute on chronic liver injury.	2013	(36)
miR-122-5p	Serum	miRCURY Exosome Isolation Kit	Acute heart failure: 42 (Japan)	n.a.	n.a.	Reflect liver damage in acute heart failure patients.	2017	(44)
miR-122, miR-200a	Serum	Exo-Quick qRT-PCR	Liver case: 13 Controls: 25 [from SMART (45) and ESPRIT (46) randomized international trials]	n.a.	n.a.	Serve as biomarkers for fatal liver disease in ART-treated, HIV-1-infected individuals.	2017	(47)
NOX1 mRNA, lncRNA ZSCAN16-AS1	Serum	Exo-Quick qRT-PCR	HC: 21 CHB: 29 HBV-ACLF: 21 (China)	n.a.	n.a.	Increase in HBV-ACLF and HBV patients compared to HC.	2020	(48)
lncRNA NEAT1	Serum	Exo-Quick qRT-PCR	ACHBLF: 185 (1) Training cohort: survivors: 54, non-survivors: 59; (2) Validation cohort: survivors:31, non-survivors:41. (China)	88.14/77.78	lncRNA NEAT1: 0.87 MELD: 0.73	A better prognostic biomarker than MELD score for 90-day mortality of ACHBLF.	2021	(49)
(1) surface ALB^+^ and VEGF^+^ (2) surface CD63^+^ and ALB^+^	Plasma	Ultracentrifugation FCM	NC: 20, CHB: 22, ACLF: 42. ACLF: survival:24, death:20. (China)	AFP: 50/80 CD63+ALB: 58.3/70 ALB+CD63+VEGF: 62.5/90	AFP: 0.64, CD63+ALB: 0.59 ALB+CD63+VEGF: 0.82	(1) Surface ALB^+^ and VEGF^+^: a more accurate and specific biomarker of liver regeneration and prognostic valuation than AFP in patients with ACLF. (2) Surface CD63^+^ and ALB^+^: an early-warning marker in patients with ACLF.	2021	(50)

ACHBLF, Acute-on-chronic hepatitis B liver failure; ACLF, acute-on-chronic liver failure; AFP, alpha-fetoprotein; ALB, albumin; ALT, alanine transaminase; ART, anti-retroviral therapies; AST, aspartate aminotransferase; AUROC, area under the receiver operating characteristic curve; CHB, chronic hepatitis B; FACS, fluorescence activated cell sorting; FCM, flow cytometry; HBV, hepatitis B virus; HC, healthy control; HIV, Human immunodeficiency virus; IL-6, interleukin-6; lncRNA NEAT1, long noncoding RNA nuclear-enriched abundant transcript 1; MELD, model for end-stage liver disease; MPs, Microparticles; NC, normal controls; NOX1, NADPH oxidase 1; qRT-PCR, quantitative real-time polymerase chain reaction; Se, sensitivity; Spe, specificity; VEGF, vascular endothelial growth factor.

#### 3.2.1 EVs as potential biomarkers for early warning or diagnosis of liver failure

##### 3.2.1.1 Preclinical studies

Conde-Vancells J et al. investigated the proteome of urinary vesicles derived from D-GalN-treated rats and attempted to identify potential biomarkers for acute liver injury (ALI). They found several proteins normally present in urinary vesicles including CD26, SLC3A1 and CD81 were dramatically reduced in urinary samples obtained from D-GalN-treated rats. And the authors believed that these three proteins had the potential to be used as candidate non-invasive urinary indicators of acute liver damage (35). CD133 and CD39 are expressed by hematopoietic stem cells (HSCs) and mobilized after liver injury. In APAP-induced experimental ALI, HSC and plasma CD133^+^ MPs levels were increased in a CD39-dependent manner. And differentially increased plasma CD39^+^ CD133^+^ MPs were helpful for monitoring critically ill patients with hepatic dysfunction and identifying patients who urgently needed for liver transplantation (36). In IRI, platelet- and neutrophil-derived MPs were acutely elevated following injury, and could serve as markers of inflammatory injury. In contrast, MPs derived from endothelial cells increased after injury response during the reparative phase, suggesting angiogenesis in the regenerating liver. Hence, MPs might be regarded as markers of acute inflammatory injury or regeneration in IRI (37). Eva Rodríguez-Suárez et al. analyzed the proteome of EVs derived from primary hepatocytes, and they found CES3, SLC27A2, HSP90, HSP70, FRIL1, CPS1, MAT and COMT increased in D-GalN-induced ALI through *in vivo* and *in vitro* experiments (38). Some unique proteins in EVs reflect the identity and tissue-specific origin of EVs. For instance, liver-specific proteins such as CES1, ADH1, GST, APOA1, ALB, HP and FGB in the EVs increased after hepatotoxin-induced liver injury. And ALB and HP in the circulating EVs were also confirmed to be increased in the alcohol-induced liver injury of the rodent model (39). Cytochrome P450 2D1 was also upregulated in hepatic EVs after GalN-induced injury, and this hepatocyte-specific enzyme could serve as a novel candidate marker to specifically detect and follow DILI (41).

Recently, exosomal miRNAs have emerged as promising biomarkers with diagnostic value. Receiver operating characteristic (ROC) analysis revealed exosomal miR-122a-5p exhibited superior diagnostic performance with an earlier diagnostic potential and a wider diagnostic time window compared to the corresponding serum counterpart in two animal models of ALI. In addition, exosomal miRNAs showed a higher correlation with ALT activity. Notably, exosomal miRNAs-122a-5p, 192-5p and 193a-3p manifested an injury-specific signature in ALI, and could be used not only as diagnostic tools but also to differentiate between different etiologies of hepatic injury (42). Furthermore, the levels of liver-specific miRNAs such as miR-122, miR-192 and miR-155 in circulating Exos were reported to be elevated in APAP-induced liver injury, but significantly decreased and returned to basal levels after treatment with antioxidant N-acetyl-cysteine (NAC), suggesting the levels of exosomal miR-122, miR-192 and miR-155 mirrored the severity of hepatocyte damage and might be used as potential sensitive diagnostic biomarkers for liver injury. High levels of circulating miRNAs are produced within certain cells in a tissue-specific manner, making them good candidate biomarkers for particular types of tissue injury. In this regard, nine miRNAs were identified as signatures of ALI. Of which, five miRNAs (miR-21a-5p, miR-92a-3p, miR-194-5p, miR-17-5p and miR-19b-3p) were increased, four miRNAs (miR-451a, miR-27a-3p, miR-26a-5p and miR-223-3p) were decreased (43).

##### 3.2.1.2 Clinical studies

ACLF is defined as an acute deterioration of liver function in patients with chronic liver diseases. It is a life-threatening clinical syndrome with a high mortality of 50–90%. Early diagnosis and recognition of patients who will die without liver transplantation are vitally important. Chen JJ and colleagues investigated differentially expressed messenger RNAs (mRNAs), long noncoding RNAs (lncRNAs), and circular RNAs (circRNAs)in circulating Exos from patients with ACLF using RNA sequencing. They found higher lncRNA but less circRNA was expressed in HBV-ACLF patients. NADPH oxidase 1 (NOX1) mRNA and lncRNA ZSCAN16-AS1 were highly expressed in patients with HBV-ACLF, and the expression levels of them were positively correlated with the progression or severity of liver injury (48). In addition, CD39^+^CD133^+^ MPs were elevated in patients with acute-on-chronic liver decompensation, suggesting acute liver insults and/or acute deterioration of liver function in the setting of chronic liver injury (36).

#### 3.2.2 EVs as potential biomarkers for prognostic assessment of liver failure

A prospective study evaluated the predictive value of serum exosomal long noncoding RNA nuclear-enriched abundant transcript 1 (LncRNA NEAT1) for 90-day mortality in acute-on-chronic hepatitis B liver failure (ACHBLF). The results displayed that lncRNA NEAT1 levels were higher in non-survivors than survivors. In the training cohort, lncRNA NEAT1 was an independent predictor for 90-day mortality of ACHBLF. Meanwhile, lncRNA NEAT1 showed a significantly higher area under the curve of receiver operating characteristic (AUC) than the MELD score in the training and validation cohort. ACHBLF patients with lncRNA NEAT1 levels above 1.92 showed poorer survival conditions than those below. Thus, serum exosomal lncRNA NEAT1 might be a better prognostic biomarker than the MELD score for 90-day mortality in ACHBLF (49). On the other hand, the assessment of liver regeneration is particularly critical for predicting prognosis and improving the quality of life in ACLF patients. Jiao Y et al. reported that the percentage of Exos with ALB, CD63 and VEGF increased in CHB, but decreased in ACLF. Among ACLF patients, the Exos with ALB, CD63 and VEGF were significantly more in the survival group than the dead group. The sensitivity and specificity of Exos with CD63, ALB and VEGF were significantly higher than other markers of liver regeneration and prognostic valuation including AFP. Therefore, the Exos with ALB and VEGF might be more accurate and specific biomarkers of liver regeneration and prognostic valuation than AFP in patients with ACLF (50).

#### 3.2.3 EVs as potential biomarkers for reflecting the severity of hepatic damage in other diseases

In some cases, liver disease is one of the main contributors to the increased morbidity and mortality in other severe diseases. Under these circumstances, circulating miRNAs might be used to reflect liver damage and develop risk assessment. For example, a prospective, observational study reported serum miR-122-5p levels were significantly positively correlated with serum liver function markers in the setting of acute heart failure (44). In anti-retroviral therapies (ART)-treated, HIV-1-infected individuals, circulating levels of miR-122 and miR-200a were elevated in HIV/HCV co-infected individuals, compared to HIV mono-infected individuals. Especially, higher pre-ART levels of circulating miR-122 and miR-200a were noticed in HIV-1 positive individuals who died from liver-related diseases whilst undergoing suppressive ART, compared to matched controls. Thus, circulating miR-122 and miR-200a were considered as promising predictive biomarkers for severe liver disease in the ART-treated, HIV-1-infected populations (47).

### 3.3 EVs as the potential treatment option for liver failure/injury

**Tables 3**, **4** listed the studies which exhibited the therapeutic potential of EVs for liver failure or liver injury induced by hepatotoxic drugs and surgery (mainly hepatic IRI), respectively. EVs derived from stem cells were most frequently reported to have therapeutic potential for liver failure or liver injury, including EVs from the human umbilical cord (blood) mesenchymal stem cells [hUC(B)MSCs], adipose-derived stem cells [A(D)SCs], and bone marrow-derived mesenchymal stem cells (BM-MSCs). Moreover, EVs derived from human menstrual blood-derived stem cells (MenSCs) (54), human-induced pluripotent stem cell-derived MSCs (hiPSC-MSCs) (67, 68) and human liver stem cells (HLSCs) (82) were also documented to protect against liver failure. Other sources of EVs included: hepatocytes (65, 66, 83), dendritic cells (DCs) (71), normal or damaged liver tissues (64) and red blood cells (RBCs) (90, 92).

**Table 3:** The therapeutic potential of EVs for liver failure/injury induced by hepatotoxic drugs.

Type of EVs	Functional cargoes	Dose of EVs	Isolation and quantification method of EVs	Animal model	Mechanism	Function	Year	Ref.
MSC-Exos	–	0.4 µg/100 µL *via* intrasplenic injection	HPLC; n.a.	C57BL/6, CCl_4_(3% vol/vol) 0.05 mL/kg i.p.	Through activation of proliferative and regenerative responses.	Elicit hepatoprotective effects against CCl_4_-induced injury.	2014	(51)
MSC-Exos	–	10 µg/100 µL, three times i.v.	Ultracentrifugation; BCA Protein Assay kit	C57BL/6, Con A 15 mg/kg i.v.	Show immunosuppressive effect.	Alleviate Con A-induced liver injury to the same extent as MSC.	2016	(52)
MSC-EVs	lncRNA Y-RNA-1	2×10^8^ to 2×10^10^ particles/body i.p./i.v.	Ultracentrifugation; NTA	C57BL/6, D-GalN 20 mg/body + TNF-α 0.3 ug/body i.p.	lncRNA Y-RNA-1 is enriched in MSC-EVs and protect hepatocyte from apoptosis.	Reduce hepatic injury and modulate cytokine expression, and improve survival from lethal hepatic failure in mice.	2017	(53)
MenSC-Exos	–	50 µg i.v.	ExoQuick-TC Precipitation kit (System Biosciences);	C57BL/6, D-GalN 800 mg/kg + LPS 50 µg/kg i.p.	Reduced the number of liver MNCs and the amount of the active apoptotic protein caspase-3 in injured livers.	Alleviate fulminant hepatic failure.	2017	(54)
hUCMSC-Exos	GPX1	16 - 32 mg/kg i.v. or oral gavage	Ultracentrifugation; BCA Protein Assay kit	Balb/C, 10% CCl_4_ 0.25 mL/kg i.p.	Reduce hepatic ROS and inhibit oxidative stress-induced apoptosis *via* upregulation of ERK1/2 and Bcl-2 and downregulation of the IKKB/NF-kB/casp-9/-3 pathway by delivery of GPX1.	Promote the recovery of hepatic oxidant injury.	2017	(55)
hUCMSC-Exos	–	6×10^10^ particles/kg i.v.	Ultracentrifugation and sucrose purification; NTA	Balb/C, 10% CCl_4_ 0.3 mL/kg, twice for an interval of 3 days i.p.	Reduced oxidative stress and inhibited apoptosis.	Present more distinct antioxidant and hepatoprotection than DDB.	2018	(56)
AMSC-Exos	miR-17	400 µg/300μL i.v.	Exosome isolation reagent (Thermo Fisher); BCA Protein Assay kit	C57BL/6, (1) D-GalN 400 mg/kg + LPS 10μg/kg; (2) D-GalN 400 mg/kg + TNF-α 20 µg/kg	By miR-17-mediated reduction of TXNIP/NLRP3 inflammasome activation in macrophages.	Ameliorate ALF induced by D-GalN/LPS.	2018	(57)
hASC-EVs	lncRNA H19	20 µg/rat or 100 µg/rat	ExoQuick-TC Precipitation kit (System Biosciences); BCA Protein Assay kit	SD rats, (1) D-GalN 0.8 g/kg, twice with a 12hour interval i.p.; (2) D-GalN 0.8 g/kg + LPS 5 µg/kg i.p.	Upregulate the HGF/c-Met pathway and related downstream channels by lncRNA H19.	Promote cell proliferation and reduce apoptosis in ALF.	2018	(58)
MSC-derived exosome-rich fractionated secretome	–	50 µg/100 µL *via* HPV	Ultracentrifugation; BCA Protein Assay kit	Wistar rats, (1) 20% CCl_4_ 5 mL/kg i.p. (2) IRI subsequent to PH.	Confer antiapoptotic and/or pro-survival effects as well as antioxidative effects *in vitro.*	Improved liver regeneration and recovery from liver injury in two models for liver failure.	2018	(59)
hUCMSC-Exos	–	100 µg/250 µL i.v.	Ultracentrifugation; BCA Protein Assay kit	C57BL/6, D-GalN 150 mg/kg + LPS 5 mg/kg i.p.	By reducing the activity of the NLRP3 inflammasome in macrophages.	Protect against ALF resulting from LPS/D-GalN.	2019	(60)
TNF-α-stimulated hUCMSC-Exos	miR-299-3p	100 mg i.v.	Ultracentrifugation; BCA Protein Assay kit	C57BL/6, D-GalN 150 mg/kg + LPS 5 mg/kg i.p.	Reduce the activation of NLRP3 in macrophages by inhibiting the disintegration of the TGN.	Attenuate inflammatory damage caused by ALF and promote liver tissue repair.	2020	(61)
hUCMSC-Exos	miR-455-3p	miR-455-3p agomir or agomir NC	ExoQuick ULTRA EV isolation kit (SBI); n.a.	C57BL/6, 10% CCl_4_ 10 mL/kg or LPS 3 mg/kg i.p.	Inhibit macrophage activation by targeting PI3K signaling.	Ameliorate IL-6-induced ALI.	2020	(62)
hUCMSC-Exos	–	20 mg/kg i.v.	Ultracentrifugation; BCA Protein Assay kit	C57BL/6, APAP 380 mg/kg i.p.	Inhibit oxidative stress-induced apoptosis *via* upregulation of ERK1/2 and PI3K/AKT signaling pathways.	Offer antioxidant hepatoprotection against APAP-induced ALF.	**2021**	(63)
*in vivo* liver EVs	–	5 µg, 4 times at 24h intervals i.v.	Ultracentrifugation and iodixanol purification; Bradford dye assay	C57BL/6, 50% CCl_4_ 2 mL/kg i.p.	Through the induction of HGF at the site of the injury by activating HSCs.	Both normal and damaged liver EVs accelerate the recovery of liver tissue from CCl_4_-induced hepatic necrosis.	2021	(64)
Hepatocyte- EVs	–	30 µg i.v.	Ultracentrifugation; BCA Protein Assay kit	C57BL/6, CCl_4_ 2 mL/kg, subcutaneously inject.	By inhibiting the recruitment of monocytes through the downregulation of chemokine receptors in the bone marrow and the recruitment of neutrophils through the reduction of CXCL1 and CXCL2 expression levels in the liver.	Attenuate the CCl_4_-induced ALI.	2021	(65)

ALF, acute liver failure; ALI, acute liver injury, AMSC, adipose tissue-derived MSC; APAP, acetaminophen; Bcl2, B-cell lymphoma 2; CCl_4_, carbon tetrachloride; Con A, concanavalin A; CXCL1, Chemokine (C-X-C motif) ligand 1; DDB, bifendate; D-GalN, D-galactosamine; ERK1/2, extracellular signal-regulated kinase 1/2; EVs, extracellular vesicles; Exos, exosomes; GPX1, glutathione peroxidase1; hASC, human adipose-derived stem cell; HFD, high-fat diet; HGF, hepatocyte growth factor; HPLC, high-performance liquid chromatography; HPV, hepatic portal vein HSCs, hepatic stellate cells; hUCMSC, human umbilical cord MSC; IL-6, interleukin-6; i.p., intraperitoneal injection; i.v., intravenous injection; lncRNA, long-chain non-coding RNA; LPS, lipopolysaccharide; MenSC, Human menstrual blood-derived stem cell; MNC, mononuclear cell; MSC, mesenchymal stem/stromal cell; n.a., not available; NC, Normal controls; NLRP3, nucleotide-binding oligomerization domain-like receptor family pyrin domain-containing 3; NTA, nanoparticle tracking analysis;PI3K, phosphoinositide 3 kinase; ROS, reactive oxygen species SD, Sprague Dawley; TGN, trans-Golgi network; TNF-α, tumor necrosis factor-α; TXNIP, thioredoxin-interacting protein.

**Table 4:** The therapeutic potential of EVs for liver failure/injury induced by surgery.

Type of EVs	Functional cargoes	Dose of EVs	Isolation and quantification method of EVs	Animal model	Mechanism	Function	Year	Ref.
Hepatocyte- Exos	neutral ceramidase and SK2	200 µg twice i.v.	Ultracentrifugation, sucrose purification and Exoquick (System Biosciences); n.a.	C57BL/6, IRI model PH model	Via increasing synthesis of S1P in target hepatocytes.	Promote hepatocyte proliferation and liver regeneration.	2016	(66)
hiPSC-MSC-Exos	–	600 µg/400μL *via* the inferior vena cava	Ultracentrifugation; BCA Protein Assay kit	SD rats, IRI model	Via suppression of inflammatory responses, attenuation of oxidative stress and inhibition of apoptosis.	Alleviate hepatic IRI.	2016	(67)
hiPSC-MSC-Exos	–	2.5 × 10^12/^500μL *via* the inferior vena cava	Ultracentrifugation; NTA	C57BL/6, IRI model	Activate SK and S1P pathway and promote hepatocyte proliferation.	Alleviate hepatic IRI.	2017	(68)
ADMSC-Exos and melatonin	–	Exos: 100 µg i.v. Melatonin: 20 - 50 mg/kg, three times i.p.	Ultracentrifugation; Coomassie blue for analysis	SD rats, IRI model	Suppress inflammation, immune cell infiltration, apoptosis, oxidative stress, DNA damage and mitochondrial damage, while promoting anti-oxidation.	Combined ADMSC-Exos and melatonin treatment are superior to either alone in protecting the liver against IRI.	2017	(69)
MSC-EVs	–	2 × 10^10/^200 µL i.v.	Ultracentrifugation; NTA	C57BL/6, IRI model	Increase Nlrp12 and CXCL1 expression, and reduce several inflammatory cytokines.	Ameliorate hepatic IRI through modulation of the inflammatory response.	2017	(70)
H/R-DEXs	HSP70	10 µg/100 µL i.v.	Ultracentrifugation; n.a.	C57BL/6, IRI model	H/R-DEXs transport HSP70 into naïve T cells and stimulate the PI3K/mTOR axis.	Improve hepatic IRI by modulating the balance of Treg and Th17 cells.	2018	(71)
hUCBMSCs-Exos	miR-1246	2.5 × 10^12/^500 µL *via* portal vein	Ultracentrifugation; NTA	C57BL/6, IRI model	MiR-1246 activated the Wnt/β-catenin signaling pathway *via* targeting GSK3β.	Alleviate hepatic IRI.	2019	(72)
hUCBMSCs-Exos	miR-1246	10 µg/100 µL i.v.	Exosome isolation kit (Invitrogen); n.a.	C57BL/6, IRI model	Via miR-1246-mediated IL-6-gp130-STAT3 axis.	Attenuate hepatic IRI by modulating Th17/Treg balance.	2019	(73)
MSC-EVs	–	1 × 10^9/^200 µL *via* the inferior vena cava	Ultracentrifugation; NTA	C57BL/6, IRI model	Reduce hepatic necrosis, increased the amount of Ki67-positive hepatocytes and repressed the transcription of inflammation-associated genes.	Have the potential to attenuate liver damage and improve regeneration after hepatic IRI.	2019	(74)
Transfected ADMSC-Exos	miR-148a	500 µg/1 mL i.v.	Ultracentrifugation; n.a.	SD rats, IRI model	Target CaMKII and regulate the Ca2+/CaMKII and TLR4 signaling pathways.	Reduce inflammatory response and apoptosis in rat hepatic IRI.	2019	(75)
hUCMSC-EVs	MnSOD	10 mg/kg i.v.	Ultracentrifugation; BCA Protein Assay kit	SD rats, IRI model	Suppress oxidative stress and neutrophil inflammatory response.	Alleviate rat hepatic IRI.	2019	(76)
hUCMSC‐Exos	miR‐20a	–	Ultracentrifugation; BCA Protein Assay kit	SD rats, IRI model	MiR‐20a binds to Beclin‐I and FAS to exert an inhibitory effect.	Restore abnormal expression of apoptosis‐ and autophagy‐related genes in I/R.	2019	(77)
hUCMSC-EVs	CCT2	100 µg/100 µL i.v.	Ultracentrifugation; BCA Protein Assay kit	C57BL/6, IRI model	Suppress CD154 expression of CD4^+^ T cells through the Ca2^+^-calcineurin-NFAT1 signaling pathway by targeting Orai1.	Attenuate liver IRI.	2020	(78)
MSC‐Heps‐Exos	–	100 µg before and after the operation i.v.	Exosome Isolation Kit (Invitrogen); Bradford method	C57BL/6, IRI model	Enhance autophagy.	Reduce hepatic IRI.	2020	(79)
MSC-Exos and GA	–	MSC- Exos: n.a., GA: 100 mg/kg i.p.	Exosome extraction kit (Thermo Fisher); n.a.	SD rats, IRI model	By maintaining the proportion of different subgroups of peripheral blood cells and restoring the expression of dysregulated proteins associated with inflammation.	GA reinforces the therapeutic effects of MSC-Exos against acute liver IRI.	2020	(80)
hUCBMSCs-Exos	miR-124	100 µg/100 µL i.v.	Ultracentrifugation and sucrose purification; n.a.	SD rats, PH model	Via downregulating Foxg1.	Promote liver regeneration and ameliorate liver injury.	2021	(81)
HLSC-EVs	–	3 × 10^9^ particles i.v.	Ultracentrifugation; NTA	C57BL/6, IRI model	By preserving tissue integrity and by reducing transaminase release and inflammatory cytokines expression.	3×10^9^ HLSC-EVs protect the liver from IRI, but a higher dose (7.5×10^9^) was ineffective, suggesting a restricted window of biological activity.	2021	(82)
Hepatocyte- EVs	Surface CD47^+^	100 µg/kg	EV isolation kit (SmartSEC Mini EV Isolation System) and ultracentrifugation; Micro BCA Protein Assay Kit	C57BL/6 Balb/C, IRI model	CD47^+^ EVs bind to CD172α on the surface of DCs, which inhibits DC activation and the cascade of inflammatory response.	CD47-enriched EVs are released in a YAP-dependent manner by hepatocytes and ameliorate hepatic IRI.	2021	(83)
ADSC-Exos	–	100 µg/600 µL i.v.	Ultracentrifugation; n.a.	SD rats, IRI subsequent to PH	By regulating mitochondrial dynamics and biogenesis.	Alleviate liver IRI subsequent to hepatectomy in rats.	2021	(84)
ADSC-Exos	PGE2	30 µg/50 µL *via* the portal vein	Ultracentrifugation; BCA Protein Assay kit	SD rats, IRI model	Via ERK1/2 and GSK-3β signaling pathways.	ADSC-Exos pre-treatment is effective in protecting liver IRI.	2022	(85)

ADMSC, adipose-derived mesenchymal stem cell; ADSC, adipose-derived stem cell; CaMKII, Ca2+/calmodulin-dependent protein kinase II; CCT2, Chaperonin containing TCP1 subunit 2; CXCL1, Chemokine (C-X-C motif) ligand 1; ERK, extracellular receptor kinase; EVs, extracellular vesicles; Exos, exosomes; GA, glycyrrhetinic acid; gp130, glycoprotein 130; GSK3β, glycogen synthase kinase 3β; hiPSC-MSC, human-induced pluripotent stem cell–derived mesenchymal stromal cell; HLSC; Human liver stem cell; H/R-DEXs, exosomes produced by bone marrow-derived dendritic cells exposed to hypoxia and reoxygenation (H/R); Hsp70, heat shock protein 70; hUCBMSC, human umbilical cord blood MSC; hUCMSC, human umbilical cord MSC; IL-6, interleukin-6; i.p., intraperitoneal injection; IRI, ischemia-reperfusion injury; i.v., intravenous injection; MnSOD, manganese superoxide dismutase; MSC, Mesenchymal stem cells; MSC‐Heps, MSC‐derived hepatocyte‐like cell; mTOR, mammalian/mechanistic target of rapamycin; n.a., not available;Nlrp12, NACHT, LRR and PYD domains-containing protein 12; NTA, nanoparticle tracking analysis;PGE2, prostaglandin E2; PH, partial hepatectomy; PI3K, phosphoinositide 3 kinase; ROS, reactive oxygen species; S1P, sphinganine-1-phosphate; SD, Sprague Dawley; SK, sphingosine kinase; STAT3, signal transducer and activator of transcription 3; Th17, T helper 17 cells; TLR4; Toll-like Receptor 4; Tregs, regulatory T cells; YAP, Yes-associated protein.

The therapeutic effects of EVs on liver failure were investigated only using rodent models. Overall, different EV therapies were documented to alleviate liver damage and improve the survival of animals.

#### 3.3.1 EVs as the potential treatment option for drug-induced liver failure/injury

Hepatotoxins D-GalN plus LPS were most often utilized to induce liver failure. Exos derived from hUCMSC with or without TNF-α treatment were reported to alleviate D-GalN/LPS-induced ALF and promote the repair of damaged liver tissues by reducing the activity of the NLRP3 inflammasome in macrophages (60, 61). miR-17 or lncRNA H19 in ASC-derived Exos protected animals from ALF by inhibiting TXNIP/NLRP3 inflammasome activation in macrophages or promoting hepatocyte proliferation mediated by the HGF/c-Met pathway (57, 58). Exos derived from BM-MSCs or human MenSCs were also demonstrated to dramatically improve the survival of mice with lethal hepatic failure by suppressing cell apoptosis (53, 54). Unexpectedly, some foods can also secrete exosome-like nanoparticles (ELNs) or vesicle-like nanoparticles (VLNs) which have protective roles. For example, shiitake mushroom-derived ELNs and honey- or garlic chive-derived VLNs were verified to protect mice against D-GalN/LPS-induced liver failure through inhibiting NLRP3 inflammasome activation (86–88) (**Table 5**).

**Table 5:** The therapeutic potential of vesicle-like nanoparticles derived from natural plants.

Type of EVs	Functional cargoes	Dose of EVs	Isolation and quantification method of EVs	Animal model	Mechanism	Function	Year	Ref.
S-ELNs		1 × 10^10/^g i.p.	Ultracentrifugation; NTA	C57BL/6, D-GalN 500 mg/kg + LPS 15 µg/kg i.p.	Inhibit NLRP3 inflammasome activation and IL-6 release.	As new inhibitors of the NLRP3 inflammasome, represent a promising class of agents with the potential to combat FHF.	2020	(86)
H-VLNs	miR-4057	0.3 × 10^10/^g, i.p.	Ultracentrifugation and sucrose purification; NTA	C57BL/6, D-GalN 500 mg/kg + LPS 15 µg/kg i.p.	Inhibit NLRP3 inflammasome activation.	Anti-inflammatory VLNs, as a new bioactive agent in honey alleviate ALI.	2021	(87)
GC-VLNs	DLPC	1 × 10^10^/g, i.p.	Ultracentrifugation and sucrose purification; NTA	C57BL/6, D-GalN 500 mg/kg + LPS 15 µg/kg i.p.	Inhibit NLRP3 inflammasome activation.	Alleviate ALI.	2021	(88)

ALI, acute liver injury; D-GalN, D-galactosamine; DLPC, 1,2-dilinoleoyl-sn-glycero-3-phosphocholine; EVs, extracellular vesicles; FHF, fulminant hepatic failure; GC, garlic chive; H-VLNs, vesicle-like nanoparticles-in honey; IL-6, interleukin-6; i.p., intraperitoneal injection; LPS, lipopolysaccharide; NLRP3, nucleotide-binding oligomerization domain-like receptor family pyrin domain-containing 3; NTA, nanoparticle tracking analysis; S-ELNs, shiitake mushroom-derived exosome-like nanoparticles.

CCl_4_ is another common hepatotoxin to induce liver failure. hUCMSC-Exos were shown to relieve CCl_4_-induced liver injury through antioxidant effects (55, 56). And miR-455-3p-enriched hUCMSC-Exos were verified to ameliorate ALI through inhibiting inflammatory response by targeting PI3K signaling (62). Exos derived from BM-MSCs were elucidated to elicit the hepatoprotective effects by activating the proliferative and regenerative responses in CCl_4_-induced liver injury (51, 59). Human hepatocyte-derived EVs were documented to attenuate ALI through modulating inflammatory immune response (65). Interestingly, the administration of both normal and damaged liver EVs was proved to significantly accelerate the recovery of liver tissue from CCl_4_-induced hepatic necrosis by inducing the production of hepatocyte growth factor at the site of the injury (64).

In addition, hUCMSC-derived EVs were demonstrated to alleviate APAP-induced ALF through activating ERK and IGF-1R/PI3K/AKT signaling pathway (63). Moreover, EVs derived from BM-MSC exerted beneficial protection through immunosuppression in the Con A-induced animal model of liver injury (52).

#### 3.3.2 EVs as the potential treatment option for liver failure/injury induced by hepatic IRI

EVs and their bioactive cargoes were confirmed to protect mice or rats against hepatic failure (**Table 4**) induced by hepatic IRI. Exos derived from ADSCs attenuated hepatic IRI *via* promoting survival mediated by ERK1/2 and GSK-3β signaling pathways (85) or regulating mitochondrial dynamics and biogenesis (84). The overexpression of miR-148a in ADSC-Exos by transfection inhibited the expressions of CaMKII and TLR4 in liver ischemia-reperfusion tissues and reduced the occurrence of the inflammatory response and apoptosis (75). hUCMSC-Exos also were documented to alleviate hepatic IRI by suppressing oxidative stress and neutrophil inflammatory response (76), inhibiting Beclin1- and FAS-mediated autophagy and apoptosis (77), or modulating inflammatory immune response (78) and promoting liver regeneration by downregulating Foxg1 (81). Exosomal miR-1246 derived from hUCBMSCs exerted anti-apoptosis, pro-survival and anti-inflammatory effects by modulating the GSK3β-mediated Wnt/β-catenin signaling pathway (72) or modulating the balance between Tregs and Th17 cells (73). In addition, EVs originating from BM-MSCs were demonstrated to protect the liver against IRI through modulating inflammatory response (increased anti-inflammatory NLRP12 expression) (70), improving hepatic regeneration (74), or enhancing autophagy (79). Moreover, hiPSC-MSC-derived Exos played a protective role in IRI *via* inhibiting inflammation, apoptosis, and oxidative stress (67) or promoting cell proliferation *via* the activation of sphingosine kinase and sphingosine-1-phosphate pathway (68). Furthermore, 3×10^9^ HLSC-EVs were able to modulate hepatic IRI by preserving tissue integrity and reducing transaminase release and inflammatory cytokines expression (82). In addition to stem cell-derived EVs, EVs from other sources also were proved to exert hepatoprotection in IRI. For instance, EVs derived from DCs were documented to attenuate IRI by transporting HSP70 to naïve T cells and stimulating the PI3K/mTOR axis to modulate the balance between Treg and Th17 Cells (71). CD47-enriched EVs released by hepatocytes in a Yes-associated protein (YAP)-dependent manner ameliorated hepatic IRI through inhibiting dendritic cell activation (83).

#### 3.3.3 Modified EVs as the potential treatment option for liver failure/injury

In some cases, EVs from the parental cells are not sufficient to effectively treat diseases. In light of this, scientists are trying their best to modify EVs or combine them with other hepatoprotective agents to optimize their functions. As a result, the protective effects of EVs against liver failure enhanced after modification. For example, the therapeutic effects of ADMSC-EVs on ALI through inhibiting inflammation or rapid senescence-like response were reinforced by combined treatment with melatonin (69) or glycyrrhetinic acid (80), loading with vitamin A and quercetin (91), or even transfection with miR-148a (75). Recently, EVs from red blood cells (RBC-EVs) are considered as preferable drug delivery vehicles because of their characteristics of low immunogenicity, easy availability and liver accumulation. RBC-EVs loaded with antisense oligonucleotides (ASOs) of miR-155 (miR155-ASOs) exhibited excellent protective and therapeutic effects against ALF by regulating macrophage polarization (90). Hybridized Ce-red blood cell vesicles (Ce-ReVs), *in situ* growth of cerium oxide (Ce) nanocrystals onto nano-sized red blood cell vesicles (ReVs), showed strong reactive oxygen species (ROS) elimination. Upon further hybridization with MSC-Exos, the resulting Ce-ReMeVs conferred superior repair benefit and brought about promising therapeutic outcomes even for models with more severe inflammatory damage, including D-GalN/LPS-induced ALI model (92). Furthermore, scientists have developed a hydrogel-mediated sustained systemic delivery of MSC-EVs to improve liver regeneration in chronic liver failure (89). In addition, systemic administration of exosome-mimicking Meseomes which is composed of membranes and secretome from efficacy-potentiated MSCs alleviated tissue necrosis and promoted the recovery of liver function in ALI models induced by CCl_4_ (93) (**Table 6**).

**Table 6:** Translational application of EVs in liver failure/injury.

Type of EVs	Translation	Dose of EVs	Animal model	Target/pathway	Function	Year	Ref.
Gel-EV	ES-MSC-EVs are encapsulated within the PEG hydrogel.	350 µg i.p.	Wistar rats, CLI: TAA 200 mg/kg i.p. twice per week for 16 weeks	EV-laden hydrogels release EVs in a sustained manner over 1 month.	Show superior antifibrosis, anti-apoptosis, and regenerative effects of the EVs when delivered by the sustained systemic release to the conventional EVs.	2019	(89)
RBC‐EVs	load miR-155-ASOs into the RBC-EVs by electroporation.	100 µg ≈ 2.3 × 10^10^ particles i.v.	C57BL/6, D-GalN 400 mg/kg + LPS 100 µg/kg i.p.	Regulate macrophage polarization.	RBC-EVs loaded with miR-155-ASOs showed macrophage-dependent protective effects against ALF.	2020	(90)
ASC-Exos	loaded with vitamin A and quercetin	200 µL i.v.	10% CCl_4_ 0.4 mL/kg i.p.	Display lower b-galactosidase positive staining and lower aging-related gene expression.	Inhibit rapid senescence-like response after ALI.	2021	(91)
Ce-ReMeVs	hybridized vesicles comprising MSC-Exos and ReV with *in situ* crystallized Ce.	5 × 10^13^/kg, i.v.	Balb/C, D-GalN 700 mg/kg + LPS 50 µg/kg i.p.	Have excellent biocompatibility, high ROS-scavenging activity and repair function of highly damaged tissues.	Alleviate ALI.	2021	(92)
exosome-mimicking Meseomes	First, MSCs are primed with IFN-γ and TNF-α. Second, exosome-mimicking Meseomes are synthesized *via* one-step extrusion.	15 ng, i.v.	Balb/C, 20% CCl_4_ 7.5 mL/kg,	Exert anti-apoptotic and pro-regenerative effects.	Alleviate ALI and also resulted in the salvage of the majority of the ischemic hindlimb (> 80%) in acute hindlimb ischemia models.	2022	(93)

ALF, acute liver failure; ALI, acute liver injury; ASC, adipose mesenchymal stem cell; ASOs, antisense oligonucleotides; CCl_4_, carbon tetrachloride; Ce, cerium oxide; D-GalN, D-galactosamine; ES-MSC, embryonic stem cell-derived MSC; EVs, extracellular vesicles; IFN-γ, interferon-γ; i.p., intraperitoneal injection; i.v., intravenous injection; LPS, lipopolysaccharide; MSC. mesenchymal stem cell; PEG, polyethylene glycol; RBC‐EVs, red blood cells-derived EVs; ReVs, red blood cells vesicles; ROS, reactive oxygen species; TAA, thioacetamide; TNF-α, tumor necrosis factor-α.

### 3.4 The quality assessment results

According to the quality assessment result by the SYRCLE tool, the majority of 57 included studies were marked as “low” or “unclear” (66.7%) risk of bias due to insufficient information regarding selection method, allocation concealment, animal housing, blinding, replacement of dropout animals, and so on (**Table S2**). The risk of bias summary and risk of bias graph were shown in **Figure S1**. All the 3 case-control studies were considered as “high” quality with a cumulative NOS score of seven points or greater. The risk of bias comprehensive assessment is shown in **Table S3**. The CERQual tool revealed that the review findings reached a “high” confidence appraisal (**Table S4**). The PRISMA checklist was completed which included further details for the review scoring (**Table S6**).

## 4 Discussion

In this review, we systematically and comprehensively summarized published studies on the administration of EVs in experimental animal models or clinical studies related to liver failure, and assessed the potential of EVs as biomarkers and treatment options for liver failure. The results revealed that EVs carrying differentially expressed proteins or nucleic acids (especially surface membrane proteins and various RNAs) can be utilized not only as early warning or diagnostic biomarkers, but also to provide clues for prognostic evaluation of liver failure. In addition, EVs and their functional cargoes have been confirmed to exert important hepatoprotective effects and can be used to treat liver failure.

Emerging studies have documented that EVs with their cargoes can serve as biomarkers for early warning, early or differential diagnosis and prognosis assessment of liver diseases, such as drug-induced liver injury, viral hepatitis, non-alcoholic fatty liver disease, alcoholic liver disease, liver cirrhosis and liver cancer (18–21). Nevertheless, it remains unclear about the potential of EVs as biomarkers of liver failure. And one of the major aims of this systematic review is to clarify this issue.

Differentially expressed proteins in EVs usually reflect the functional properties and tissue-specific origin of EVs, as well as underlying pathophysiological mechanisms, thus they have the potential to act as emerging biomarkers for early warning or diagnosis of liver failure. Multiple studies in this systematic review showed that the unique proteins, including intrinsic proteins or surface membrane proteins carried by EVs, are good early warning or diagnostic biomarkers for liver failure. In terms of preclinical studies, elevated surface protein CD133 (36) or intrinsic proteins ALB, HP and FGB (39) in circulating EVs may serve as early warning biomarkers in APAP-induced liver injury. In addition, several proteins such as CES3, SLC27A2, and HSP90 were found to be more commonly expressed in EVs isolated from sera of D-GalN-induced rats (38). On the contrary, some proteins (such as CD26, SLC3A1 and CD81) that are normally present in urinary vesicles were discovered to be dramatically reduced in urinary samples obtained from D-GalN-treated rats (35). In hepatic IRI, MPs derived from platelets (with surface CD41^+^) and neutrophils (with surface Ly-6G^+^) may be markers of hepatic inflammation following injury, and MPs derived from endothelial cells (with surface CD62E^+^) may reflect angiogenesis during the reparative phase (37).

In addition, differentially expressed RNA, especially miRNA in EVs, can also be considered as early warning, diagnosis or prognosis evaluation biomarkers of liver failure. For instance, miR-122, miR-192 and miR-155 carried by EVs may serve as biomarkers of ALI considering that they were upregulated in APAP-induced liver injury and reversed to basal levels after NAC treatment (40). Notably, exosomal miR-122a-5p was regarded as the best diagnostic biomarker with higher diagnostic power and a wider diagnostic window for ALI (42). Furthermore, a set of miRNAs including miR-122-5p, miR-192-5p and miR-22-3p were considered as potential biomarkers which contribute mainly to liver steatosis and inflammation in ALI (43).

Consistent with findings in experimental animal studies, differentially expressed surface proteins and RNAs carried by EVs also may be considered as potential early warning or prognostic biomarkers for liver failure in clinical studies. ALB and VEGF carried by Exos may be more accurate and specific biomarkers of liver regeneration and prognostic evaluation than AFP in serum for patients with ACLF, and CD63^+^ALB^+^Exos may be an early warning marker for ACLF patients (50). Furthermore, NOX1 mRNA and lncRNA ZSCAN16-AS1 were reported to be increased in HBV-ACLF and HBV patients compared to healthy control (48). Especially, lncRNA NEAT1 is a better prognostic biomarker than the MELD score for 90-day mortality in ACHBLF (49). Interestingly, miR-122 has also been found to reflect the severity of liver damage in patients with other diseases, such as patients with acute heart failure and ART-treated, HIV-1-infected individuals (44, 47). However, more comprehensive and large-scale clinical studies are still needed in the further to verify the role of EVs as good biomarkers for liver failure.

Although preclinical and clinical studies have revealed that EVs may be potential early warning, diagnosis or prognosis evaluation biomarkers of liver failure, research in this area remains to be in its infancy and large-scale clinical studies are scarce. This may be ascribed to the followings: (1) there is no widely accepted ideal animal model of liver failure, especially ACLF; (2) high technical requirements and detection costs as well as uncertain results limit the implementation of studies related to Exos; (3) the etiologies of liver failure are diversified, which leads to great individual variation; (4) liver failure is often accompanied by complex complications, which may act as confounding factors and limit the diagnostic and prognostic value of EVs for liver failure in clinical studies.

The other objective of this systematic review is to assess the treatment potential of EVs in liver failure. To this end, we comprehensively summarized the studies in which EVs are utilized to treat liver failure. EVs derived from stem cells, especially MSCs, are most commonly used to treat liver failure. MSCs can be isolated from bone marrow, adipose tissue, umbilical cord, and so on. They have been documented to possess the strong ability of differentiation, regeneration and immunomodulation, and can be used to treat various liver diseases efficiently and safely in clinical studies (96, 97). The therapeutic effect of MSCs can be attributed to paracrine, especially EVs, to a large extent. Compared with MSCs, MSC-EVs have the following advantages: (1) much smaller in size and easier to pass through biological barriers including the blood-brain barrier; (2) can be administered intravenously, and more likely accumulate in the liver; (3) cryopreservation does not affect the clinical efficacy; (4) lower immunogenicity and better histocompatibility. Therefore, EVs are emerging as a novel treatment option.

EVs and their functional cargoes turn out to play important hepatoprotective effects in liver failure by inhibiting inflammatory response, oxidative stress, apoptosis or promoting hepatocyte regeneration and autophagy.

Regarding the inhibition of inflammation response, hUCMSCs-Exos containing miR-299-3p or miR-455-3p ameliorated drug-induced ALF through inhibiting the recruitment and activation of the NLRP3 inflammasomes or modulating PI3K signaling (61, 62). Moreover, hUC(B)MSCs-EVs protected hepatic IRI through suppressing CD154 expression on CD4^+^ T cells *via* CCT2 (78) or modulating the balance between Tregs and Th17 cells *via* miR-1246-mediated IL-6-gp130-STAT3 axis (73). Furthermore, AMSC-derived Exos carrying miR-17 ameliorated GalN/LPS-induced ALF by targeting TXNIP, which is well-known as a key player in the activation of NLRP3 inflammasome (57). In addition, BM-MSC-derived EVs protected against murine hepatic ischemia/reperfusion injury by increasing NLRP12 and CXCL1 expression and reducing several inflammatory cytokines such as IL-6 (70).

In the light of resistance to oxidative stress and apoptosis, hUCMSC-Exos inhibited oxidative stress-induced apoptosis in APAP- or CCl_4_-induced liver injury by upregulation of ERK and IGF-1R/PI3K/AKT signaling pathways and downregulation of the IKKB/NF-kB/casp-9/-3 pathway (55, 63, 65). ADSC-Exos protected against liver IRI by regulating mitochondrial dynamics and biogenesis or ERK1/2 and GSK3beta signaling pathways (84, 85). Moreover, MenSC-Exos reduced the number of liver mononuclear cells (MNCs) and the amount of the active apoptotic protein caspase-3 in D-GalN/LPS-induced fulminant hepatic failure (54).

EVs have also been proven to treat liver failure through promoting hepatocyte regeneration and proliferation. For example, hASCs-EVs containing lncRNA H19 promoted hepatocyte regeneration in D-GalN-induced ALF *via* upregulating the HGF/c-Met pathway (58). And hUCBMSC-Exos enriched with miR-124 promoted liver regeneration in partial hepatectomy by downregulating Foxg1 (81). Moreover, hiPSC-MSCs-derived Exos alleviated hepatic IRI through activating sphingosine kinase (SK) and sphingosine-1-phosphate(S1P) signaling pathway and promoting hepatocyte proliferation (68).

With respect to enhancement of autophagy, Exos obtained from MSC‐derived hepatocyte‐like cells treatment enhanced autophagy during hepatic IRI to exert the hepatoprotective effect (79), and hUCMSC‐Exos carried with miR‐20a inhibited Beclin‐1 and FAS to alleviate the abnormal expression of apoptosis‐ and autophagy‐related genes in liver ischemia-reperfusion (77).

In addition to stem cell-derived EVs, EVs derived from other cells or tissues have also been confirmed to protect against liver failure, such as hepatocytes (65, 66, 83), DCs (71) as well as normal or damaged liver tissue (64). Interestingly, researchers also extracted desired EVs from natural foods, such as mushroom, honey and garlic chive, and proved that they may have a protective effect on the liver by inhibiting the activation of inflammasome (86–88). These findings greatly broaden the source of EVs and provide new ideas for the treatment of liver failure.

In recent years, scientists have attempted to modify EVs to achieve superior therapeutic effects for liver failure. For example, red blood cells which have the features of low immunogenicity, easy availability and liver accumulation were transfected with hepatoprotective miRNAs [such as miR155‐ASOs (90)] or hybridized with Ce and MSC-Exos (92) to improve the targeting and therapeutic effect of EVs on ALF. Moreover, the protective effect of ADMSC-Exos on liver failure was improved by loading them with vitamin A and quercetin (91). In addition, EV-encapsulated PEG hydrogels were developed to retard the clearance of MSC-EVs, ultimately improving liver regeneration in chronic liver failure (89).

## 5 Conclusion

**Figure 2:**
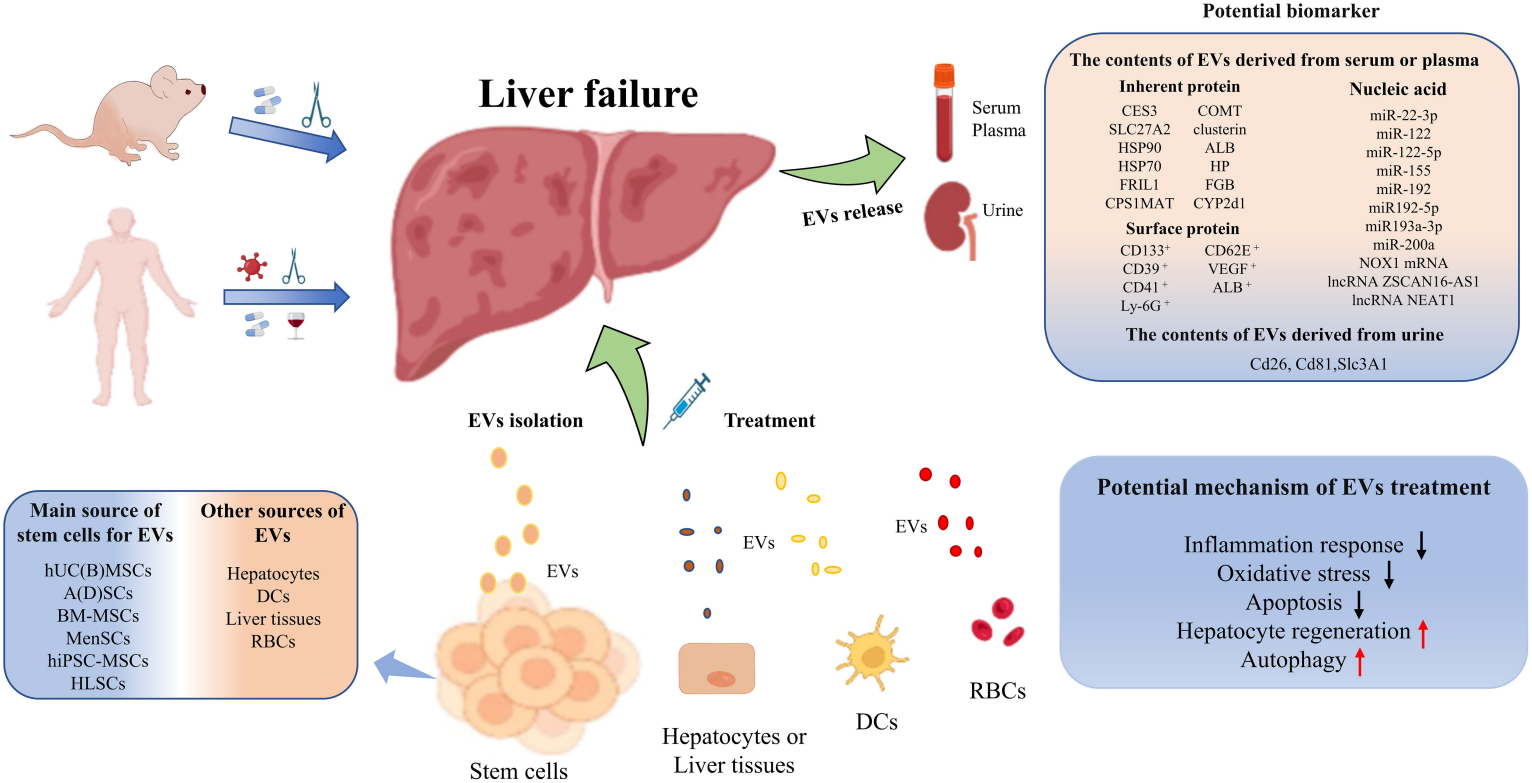
Graphical Abstract to summarize the potential of biomarkers and treatment options for EVs.

In conclusion, EVs and their cargoes can be used not only as superior biomarkers of early warning, diagnosis and prognostic assessments for liver failure, but also as potentially effective treatment options for patients with liver failure (**Figure 2**). In the future, large-scale studies are urgently needed to verify the diagnostic, predictive and therapeutic value of EVs for liver failure.

## Data availability statement

The original contributions presented in the study are included in the article/**Supplementary Material**. Further inquiries can be directed to the corresponding authors.

## Author contributions

WL and HT drafted the paper. SL provided literature search support. YC and LB revised the manuscript. All authors contributed to the article and approved the submitted version.
