# Supplementary material for: Extracellular vesicles as potential biomarkers and treatment options for liver failure: A systematic review up to March 2022
Source: Front Immunol. 2023 Feb 22;14:1116518. doi: 10.3389/fimmu.2023.1116518 (PMC9992400; doi:10.3389/fimmu.2023.1116518)
Supplement: Supplementary file 1 [file DataSheet_1.docx]

***Supplementary Material***

**Extracellular vesicles****as potential biomarkers and treatment option for liver failure: a** **systematic review**

Wang Lu^1,2†^, Huixin Tang^1,2†^, Shanshan Li^1,2^, Li Bai^1,2*^, Yu Chen^1,2*^

^†^These authors contributed equally to this work.

^*^Correspondence: Yu Chen, E-mail: chybeyond1071@ccmu.edu.cn. Li Bai, E-mail: tender78@ccmu.edu.cn.

**Table S1 PubMed search strategy**

| **Number** | **Search items** |
| --- | --- |
| #1 | "Extracellular Vesicles"[Mesh] OR Exosome*[tw] OR Extracellular Vesicle*[tw] OR Exovesicle*[tw] OR Apoptotic Body[tw] OR Apoptotic Bodies[tw] OR EVs[tw] OR Ectosome*[tw] OR "Cell-Derived Microparticle*"[tw] OR Shedding Microvesicle*[tw] OR Shedding Microvesicle*[tw] OR "Cell Membrane Microparticle*"[tw] |
| #2 | "Liver Failure"[Mesh] OR Liver Failure*[tw] OR Hepatic Failure*[tw] OR Hepatic Encephalopath*[tw] OR Hepatocerebral Encephalopath*[tw] OR Portal-Systemic Encephalopath*[tw] OR Portosystemic Encephalopath*[tw] OR Hepatic Coma*[tw] OR Hepatic Stupor*[tw] OR "Acute Yellow Atroph*"[tw] OR Hepatic Necrosis[tw] OR Hepatic Necroses[tw] OR Fulminant Hepatitis[tw] OR Fulminant Hepatitides[tw] OR Hepatic injury[tw] OR Hepatic injuries[tw] OR Liver injury[tw] OR Liver injuries[tw] |
| #3 | #1AND #2 |

**Table S2** **SYRCLE’s risk of bias tool**

|  | **Selection bias** | | | **Performance bias** | | **Detection bias** | | **Attrition bias** | **Reporting bias** | **Other** | | | | |
| --- | --- | --- | --- | --- | --- | --- | --- | --- | --- | --- | --- | --- | --- | --- |
| **Study** | **Sequence generation** | **Baseline characteristics** | **Allocation concealment** | **Random housing** | **Blinding** | **Random outcome assessment** | **Blinding** | **Incomplete outcome data** | **Selective outcome reporting** | **Free of contamination** | **Free of inappropriate influence of funders** | **Free of unit of analysis errors** | **Design-specific risk of bias** | **New animals added to replace drop-outs** |
| Conde-Vancells et al. 2010(35) | Unclear | Yes | Unclear | Unclear | No | No | No | Unclear | Yes | Unclear | Unclear | Unclear | Yes | Unclear |
| Schmelzle et al. 2013(36) | Unclear | Yes | Unclear | Unclear | No | Unclear | No | Unclear | Yes | Unclear | Unclear | Unclear | Unclear | Unclear |
| Freeman et al. 2014(37) | Unclear | Yes | Unclear | Unclear | Unclear | Unclear | Unclear | Unclear | Yes | Unclear | Unclear | Unclear | Yes | Unclear |
| Rodriguez-Suarez et al. 2014(38) | Yes | Yes | Yes | Unclear | Unclear | Unclear | Unclear | Unclear | Yes | Unclear | Unclear | Unclear | Yes | Unclear |
| Cho, Kim, et al. 2017(40) | Unclear | Yes | Unclear | Unclear | No | No | No | Unclear | Yes | Unclear | Unclear | Unclear | Yes | Unclear |
| Cho, Im, et al. 2017(39) | Unclear | Yes | Unclear | Unclear | No | No | No | Unclear | Yes | Unclear | Yes | Unclear | Yes | Unclear |
| Palomo et al. 2018(41) | Yes | Yes | Yes | Yes | Unclear | Unclear | Unclear | Unclear | Yes | Yes | Unclear | Unclear | Yes | Unclear |
| Motawi et al. 2018(42) | Unclear | Yes | Unclear | Unclear | Unclear | Unclear | Unclear | Unclear | Yes | Unclear | Unclear | Unclear | Yes | Unclear |
| Lv et al. 2021(43) | Unclear | Yes | Unclear | Unclear | Unclear | Unclear | Unclear | Unclear | Yes | Unclear | Unclear | Unclear | Yes | Unclear |
| Tan et al. 2014(53) | Unclear | Yes | Unclear | Unclear | Unclear | Unclear | Unclear | Unclear | Yes | Unclear | Unclear | Yes | Yes | Unclear |
| Tamura, Uemoto, and Tabata 2016(54) | Unclear | Yes | Unclear | Unclear | Unclear | Unclear | Unclear | Unclear | Yes | Unclear | Unclear | Unclear | Yes | Unclear |
| Haga, Yan, Takahashi, et al. 2017(55) | Unclear | Yes | Unclear | Unclear | Unclear | Unclear | Unclear | Unclear | Yes | Unclear | Unclear | Unclear | Yes | Unclear |
| Chen et al. 2017(56) | Unclear | Yes | Unclear | Unclear | Unclear | Unclear | Unclear | Unclear | Yes | Unclear | Unclear | Unclear | Yes | Unclear |
| Yan et al. 2017(57) | Yes | Yes | Yes | Yes | Unclear | Unclear | Unclear | Unclear | Yes | Unclear | Unclear | Unclear | Yes | Unclear |
| Jiang et al. 2018(58) | Yes | Yes | Yes | Yes | Unclear | Unclear | Unclear | Unclear | Yes | Unclear | Yes | Unclear | Yes | Unclear |
| Liu et al. 2018(59) | Unclear | Yes | Unclear | Unclear | Unclear | Unclear | Unclear | Unclear | Yes | Unclear | Unclear | Unclear | Yes | Unclear |
| Jin et al. 2018(60) | Yes | Yes | Yes | Yes | Yes | Yes | Yes | Yes | Yes | Yes | Unclear | Unclear | Yes | Unclear |
| Damania et al. 2018(61) | Unclear | Yes | Unclear | Unclear | Unclear | Unclear | Unclear | Unclear | Yes | Unclear | Unclear | Unclear | Yes | Unclear |
| Jiang et al. 2019(62) | Yes | Yes | Yes | Yes | Unclear | Unclear | Unclear | Unclear | Yes | Unclear | Unclear | Unclear | Yes | Unclear |
| Zhang, Jiang, et al. 2020(63) | Unclear | Yes | Unclear | Unclear | Unclear | Unclear | Unclear | Unclear | Yes | Unclear | Unclear | Unclear | Yes | Unclear |
| Shao et al. 2020(64) | Unclear | Yes | Unclear | Unclear | Unclear | Unclear | Unclear | Unclear | Yes | Unclear | Unclear | Unclear | Yes | Unclear |
| Wu et al. 2021(65) | Yes | Yes | Yes | Yes | Unclear | Unclear | Unclear | Unclear | Yes | Unclear | Yes | Unclear | Yes | Unclear |
| Lee et al. 2021(66) | Unclear | Yes | Unclear | Unclear | Unclear | Unclear | Unclear | Unclear | Yes | Unclear | Unclear | Unclear | Yes | Unclear |
| Kakizaki et al. 2021(67) | Yes | Yes | Yes | Yes | Yes | Yes | Yes | Unclear | Yes | Unclear | Unclear | Unclear | Yes | Unclear |
| Nojima et al. 2016(68) | Yes | Yes | Yes | Yes | Yes | Yes | Yes | Yes | Yes | Yes | Unclear | Unclear | Yes | Unclear |
| Nong et al. 2016(69) | Unclear | Yes | Unclear | Unclear | Unclear | Unclear | Unclear | Unclear | Yes | Unclear | Unclear | Unclear | Yes | Unclear |
| Du et al. 2017(70) | Yes | Yes | Yes | Yes | Yes | Yes | Yes | Yes | Yes | Unclear | Unclear | Unclear | Yes | Unclear |
| Sun et al. 2017(71) | Unclear | Yes | Unclear | Unclear | Unclear | Unclear | Unclear | Unclear | Yes | Unclear | Unclear | Unclear | Yes | Unclear |
| Haga, Yan, Borrelli, et al. 2017(72) | Unclear | Yes | Unclear | Unclear | Unclear | Unclear | Unclear | Unclear | Yes | Unclear | Unclear | Unclear | Yes | Unclear |
| Zheng et al. 2018(73) | Unclear | Yes | Unclear | Unclear | Unclear | Unclear | Unclear | Unclear | Yes | Unclear | Unclear | Unclear | Unclear | Unclear |
| Xie et al. 2019a(75) | Unclear | Yes | Unclear | Unclear | Unclear | Unclear | Unclear | Unclear | Yes | Unclear | Unclear | Unclear | Yes | Unclear |
| Xie et al. 2019b(74) | Unclear | Yes | Unclear | Unclear | Unclear | Unclear | Unclear | Unclear | Yes | Unclear | Unclear | Unclear | Yes | Unclear |
| Anger et al. 2019(76) | Unclear | Yes | Unclear | Unclear | Unclear | Unclear | Unclear | Unclear | Yes | Unclear | Unclear | Unclear | Yes | Unclear |
| Nong et al. 2019(77) | Unclear | Yes | Unclear | Unclear | Unclear | Unclear | Unclear | Unclear | Yes | Unclear | Unclear | Unclear | Yes | Unclear |
| Yao et al. 2019(78) | Yes | Yes | Yes | Yes | Unclear | Unclear | Unclear | Unclear | Yes | Unclear | Unclear | Unclear | Yes | Unclear |
| Zhang, Song, et al. 2020(79) | Unclear | Yes | Unclear | Unclear | Unclear | Unclear | Unclear | Unclear | Yes | Unclear | Unclear | Unclear | Yes | Unclear |
| Zheng et al. 2020(80) | Yes | Yes | Yes | Yes | Unclear | Unclear | Unclear | Unclear | Yes | Unclear | Unclear | Unclear | Yes | Unclear |
| Yang et al. 2020(81) | Unclear | Yes | Unclear | Unclear | Unclear | Unclear | Unclear | Unclear | Yes | Unclear | Unclear | Unclear | Yes | Unclear |
| Wei et al. 2020(82) | Unclear | Yes | Unclear | Unclear | Unclear | Unclear | Unclear | Unclear | Yes | Unclear | Unclear | Unclear | Yes | Unclear |
| Song et al. 2021(83) | Unclear | Yes | Unclear | Unclear | Unclear | Unclear | Unclear | Unclear | Yes | Unclear | Unclear | Unclear | Yes | Unclear |
| Yuan et al. 2021(85) | Unclear | Yes | Unclear | Unclear | Unclear | Unclear | Unclear | Unclear | Yes | Unclear | Unclear | Unclear | Yes | Unclear |
| Calleri et al. 2021(84) | Yes | Yes | Yes | Yes | Yes | Yes | Yes | Yes | Yes | Yes | Unclear | Unclear | Yes | No |
| Zhang et al. 2021(86) | Yes | Yes | Yes | Unclear | Unclear | Unclear | Unclear | Unclear | Yes | Yes | Unclear | Unclear | Yes | Unclear |
| Zhang et al. 2022(87) | Unclear | Yes | Unclear | Unclear | Unclear | Unclear | Unclear | Unclear | Yes | Unclear | Unclear | Unclear | Yes | Unclear |
| Liu et al. 2020(90) | Unclear | Yes | Unclear | Unclear | Unclear | Unclear | Unclear | Unclear | Yes | Unclear | Unclear | Unclear | Yes | Unclear |
| Chen et al. 2021(91) | Unclear | Yes | Unclear | Unclear | Unclear | Unclear | Unclear | Unclear | Yes | Unclear | Unclear | Unclear | Yes | Unclear |
| Liu et al. 2021(92) | Yes | Yes | Yes | Yes | Unclear | Unclear | Unclear | Unclear | Yes | Unclear | Unclear | Unclear | Yes | Unclear |
| Mardpour et al. 2019(94) | Unclear | Yes | Unclear | Unclear | Unclear | Unclear | Unclear | Yes | Yes | Unclear | Unclear | Unclear | Yes | Unclear |
| Zhang, Huang, et al. 2020(88) | Unclear | Yes | Unclear | Unclear | Unclear | Unclear | Unclear | Unclear | Yes | Unclear | Unclear | Unclear | Yes | Unclear |
| Fang and Liang 2021(93) | Yes | Yes | Yes | Unclear | Unclear | Unclear | Unclear | Unclear | Yes | Unclear | Unclear | Unclear | Yes | Unclear |
| Zhao et al. 2021(89) | Unclear | Yes | Unclear | Unclear | Unclear | Unclear | Unclear | Unclear | Yes | Unclear | Unclear | Unclear | Yes | Unclear |
| Qi, Liu et al. 2022(95) | Yes | Yes | Yes | Unclear | Unclear | Unclear | Unclear | Unclear | Yes | Unclear | Unclear | Unclear | Yes | Unclear |

**Figure S1 The risk of bias summary using the SYRCLE’s risk of bias tool**


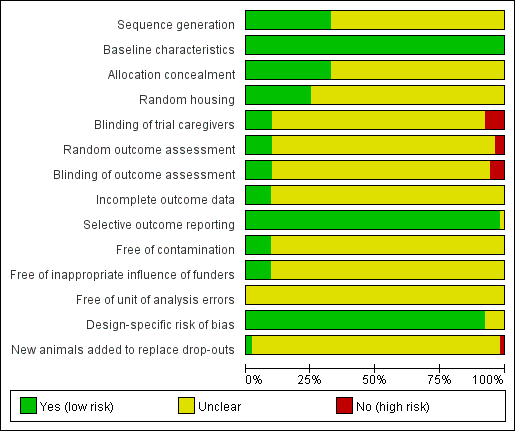


**Table S3 Evaluation of the quality of case-control studies included in the system review (assessed by Newcastle-Ottawa Scale)**

| Study | Year | Selection | | | | Comparability of cases and controls on the basis of the design or analysis | Exposure | | | Scores |
| --- | --- | --- | --- | --- | --- | --- | --- | --- | --- | --- |
|  |  | Adequate definition of cases | Representativeness of the cases | Selection of Controls | Definition of Controls |  | Ascertainment of exposure | Same method of ascertainment for cases and controls | Non-Response rate |  |
| Murray | 2017 | ★ | ★ | ★ |  | ★ | ★ | ★ | ★ | 7 |
| Koyama | 2017 | N.A. |  |  |  |  |  |  |  |  |
| Chen | 2020 | ★ |  | ★ | ★ | ★ | ★ | ★ | ★ | 7 |
| Gao | 2021 | N.A. |  |  |  |  |  |  |  |  |
| Jiao | 2021 | ★ | ★ | ★ | ★ | ★ | ★ | ★ | ★ | 8 |

**N.A.:** not applicable.

**Table S4 The evidence quality of outcomes (assessed by CERQual tool)**

| **Outcomes** | **Studies contributing to the**  **outcomes** | **Domain 1:** **the methodological limitations** | **Domain 2: the relevance** | **Domain 3: the coherence** | **Domain 4:** **the adequacy of the data** | **Overall CERQual**  **rating for assessment of confidence** |
| --- | --- | --- | --- | --- | --- | --- |
| Potential biomarker | Conde-Vancells 2010, Schmelzle 2013, Freeman 2014, Rodriguez-Suarez 2014, Cho 2017, Cho 2017, Palomo 2018, Motawi 2018, Lv et 2021, Murray 2017, Koyama 2017, Chen 2020, Gao 2021, Jiao 2021. | The methodological limitations in the domains important to internal validity in primary studies mainly exist in detection bias, reporting bias. | It is feasible to apply it in clinical trials and the limitation is mainly about the unified measurement methods and gold standards. | All 14 included studies have reported that the contents carried by EVs can be used as potential biomarkers of liver injury or liver failure. | Most of the included studies demonstrated that disease states altered EVs contents and that specific EVs contents could serve as biomarkers of disease. | ⊕⊕⊕⊕  High |
| Potential treatment option | Tan 2014, Tamura 2016, Haga 2017, Chen 2017, Yan 2017, Jiang 2018, Liu 2018, Jin 2018, Damania 2018, Jiang 2019, Zhang 2020, Shao 202, Wu 2021, Lee 2021, Kakizaki 2021, Nojima 2016, Nong 2016, Du 2017, Sun 2017, Haga 2017, Zheng 2018, Xie 2019, Xie 2019, Anger 2019, Nong 2019, Yao 2019, Zhang 2020, Zheng 2020, Yang 2020, Wei 2020, Song 2021, Yuan 2021, Calleri 2021, Zhang 2021, Zhang 2022, Liu 2020, Chen 2021, Liu 2021, Mardpour 2019, Zhang 2020, Fang 2021, Zhao 2021, Qi 2022. | The methodological limitations in the domains  important to internal validity in primary studies mainly exist in blinding for assessors and grouping randomization and concealment. | Transformation to clinical trials is feasible and the limitation is mainly about the appropriate source of EVs and standardized production and storage. | All 43 included studies reported that EVs from a variety of sources, such as multiple stem cells, DC cells, liver tissue hepatocytes and natural plants, exerted liver protective effects and were able to alleviate acute or chronic liver injury. | Most of the included studies evaluated the protective effects of EVs against liver failure or liver injury. | ⊕⊕⊕⊕  High |

**Table S5 AMSTAR 2 criteria for the systematic reviews assessed by two authors**

| **Item** | **A** | **B** |
| --- | --- | --- |
| 1. Did the research questions and inclusion criteria for the review include the components of PICO? | Yes | Yes |
| 2. Did the report of the review contain an explicit statement that the review methods were established prior to the conduct of the review and did the report justify any significant deviations from the protocol? | Yes | Yes |
| 3. Did the review authors explain their selection of the study designs for inclusion in the review? | Yes | Yes |
| 4. Did the review authors use a comprehensive literature search strategy? | Yes | Yes |
| 5. Did the review authors perform study selection in duplicate? | Yes | Yes |
| 6. Did the review authors perform data extraction in duplicate? | Yes | Yes |
| 7. Did the review authors provide a list of excluded studies and justify the exclusions? | Yes | Yes |
| 8. Did the review authors describe the included studies in adequate detail? | Yes | Yes |
| 9. Did the review authors use a satisfactory technique for assessing the risk of bias (RoB) in individual studies that were included in the review? | Yes | Yes |
| 10. Did the review authors report on the sources of funding for the studies included in the review? | No | No |
| 11. If meta-analysis was performed, did the review authors use appropriate methods for statistical combination of results? | N.A. | N.A. |
| 12. If meta-analysis was performed, did the review authors assess the potential impact of RoB in individual studies on the results of the meta-analysis or other evidence synthesis? | N.A. | N.A. |
| 13. Did the review authors account for RoB in primary studies when interpreting/discussing the results of the review? | Yes | Yes |
| 14. Did the review authors provide a satisfactory explanation for, and discussion of, any heterogeneity observed in the results of the review? | Yes | Yes |
| 15. If they performed quantitative synthesis did the review authors carry out an adequate investigation of publication bias (small study bias) and discuss its likely impact on the results of the review? | N.A. | N.A. |
| 16. Did the review authors report any potential sources of conflict of interest, including any funding they received for conducting the review? | Yes | Yes |
| **Total Yes, N (%)** | **12/13 (92)** | **12/13 (92)** |

**PICO:** Population, Intervention, Control group, and Outcome. **RoB:** Risk of Bias. **N.A.:** not applicable.

**Table S6 PRISMA checklist**

| **Section/topic** | **#** | **Checklist item** | **Reported on page #** |
| --- | --- | --- | --- |
| **TITLE** | | |  |
| Title | 1 | Identify the report as a systematic review, meta-analysis, or both. | 1 |
| **ABSTRACT** | | |  |
| Structured summary | 2 | Provide a structured summary including, as applicable: background; objectives; data sources; study eligibility criteria, participants, and interventions; study appraisal and synthesis methods; results; limitations; conclusions and implications of key findings; systematic review registration number. | 1 |
| **INTRODUCTION** | | |  |
| Rationale | 3 | Describe the rationale for the review in the context of what is already known. | 2 |
| Objectives | 4 | Provide an explicit statement of questions being addressed with reference to participants, interventions, comparisons, outcomes, and study design (PICOS). | 2 |
| **METHODS** | | |  |
| Protocol and registration | 5 | Indicate if a review protocol exists, if and where it can be accessed (e.g., Web address), and, if available, provide registration information including registration number. | 2,3 |
| Eligibility criteria | 6 | Specify study characteristics (e.g., PICOS, length of follow-up) and report characteristics (e.g., years considered, language, publication status) used as criteria for eligibility, giving rationale. | 3 |
| Information sources | 7 | Describe all information sources (e.g., databases with dates of coverage, contact with study authors to identify additional studies) in the search and date last searched. | 2,3 |
| Search | 8 | Present full electronic search strategy for at least one database, including any limits used, such that it could be repeated. | 2,3, S1 |
| Study selection | 9 | State the process for selecting studies (i.e., screening, eligibility, included in systematic review, and, if applicable, included in the meta-analysis). | 2,3,4 |
| Data collection process | 10 | Describe method of data extraction from reports (e.g., piloted forms, independently, in duplicate) and any processes for obtaining and confirming data from investigators. | 2,3,4 |
| Data items | 11 | List and define all variables for which data were sought (e.g., PICOS, funding sources) and any assumptions and simplifications made. | 2,3,4 |
| Risk of bias in individual studies | 12 | Describe methods used for assessing risk of bias of individual studies (including specification of whether this was done at the study or outcome level), and how this information is to be used in any data synthesis. | 4,6 |
| Summary measures | 13 | State the principal summary measures (e.g., risk ratio, difference in means). | N/A |
| Synthesis of results | 14 | Describe the methods of handling data and combining results of studies, if done, including measures of consistency (e.g., I^2^) for each meta-analysis. | N/A |
| **Section/topic** | **#** | **Checklist item** | **Reported on page #** |
| Risk of bias across studies | 15 | Specify any assessment of risk of bias that may affect the cumulative evidence (e.g., publication bias, selective reporting within studies). | 4,6 |
| Additional analyses | 16 | Describe methods of additional analyses (e.g., sensitivity or subgroup analyses, meta-regression), if done, indicating which were pre-specified. | N/A |
| **RESULTS** | | |  |
| Study selection | 17 | Give numbers of studies screened, assessed for eligibility, and included in the review, with reasons for exclusions at each stage, ideally with a flow diagram. | 6 |
| Study characteristics | 18 | For each study, present characteristics for which data were extracted (e.g., study size, PICOS, follow-up period) and provide the citations. | 6~9 |
| Risk of bias within studies | 19 | Present data on risk of bias of each study and, if available, any outcome level assessment (see item 12). | 13 |
| Results of individual studies | 20 | For all outcomes considered (benefits or harms), present, for each study: (a) simple summary data for each intervention group (b) effect estimates and confidence intervals, ideally with a forest plot. | N/A |
| Synthesis of results | 21 | Present results of each meta-analysis done, including confidence intervals and measures of consistency. | N/A |
| Risk of bias across studies | 22 | Present results of any assessment of risk of bias across studies (see Item 15). | N/A |
| Additional analysis | 23 | Give results of additional analyses, if done (e.g., sensitivity or subgroup analyses, meta-regression [see Item 16]). | N/A |
| **DISCUSSION** | | |  |
| Summary of evidence | 24 | Summarize the main findings including the strength of evidence for each main outcome; consider their relevance to key groups (e.g., healthcare providers, users, and policy makers). | 13~15 |
| Limitations | 25 | Discuss limitations at study and outcome level (e.g., risk of bias), and at review-level (e.g., incomplete retrieval of identified research, reporting bias). | 13~15 |
| Conclusions | 26 | Provide a general interpretation of the results in the context of other evidence, and implications for future research. | 15 |
| **FUNDING** | | |  |
| Funding | 27 | Describe sources of funding for the systematic review and other support (e.g., supply of data); role of funders for the systematic review. | 15 |

**N/A:** not available. **S1:** **Table S1.**
